# IRX1 hypomethylation in osteosarcoma metastasis

**DOI:** 10.18632/oncotarget.4696

**Published:** 2015-06-30

**Authors:** Jinchang Lu, Jin Wang

**Affiliations:** Department of Musculoskeletal Oncology, the First Affiliated Hospital of Sun Yat-Sen University, Guangzhou, Guangdong, China

**Keywords:** osteosarcoma, IRX1, DNA methylation, metastasis, ctDNA

Osteosarcoma, the most common primary bone cancer in children and adolescents, is notorious for its potential to metastasize to lungs at the very early stage. Despite improvements in both chemotherapy and surgical managements in last two decades, the five-year survival rate remains at only 20% in metastatic patients. Thus, identifying biomarkers for early detection of metastasis and developing novel therapeutic approaches are urgently needed to improve survival.

DNA methylation, which involves covalent addition of a methyl group in cytosine within CpG dinucleotides, is an important epigenetic mechanism that regulates the gene expression. Aberrant DNA methylation-mediated gene silencing and activation can affect almost all the cellular signaling pathways that participate in tumor progression. Unlike genetic changes, methylation alterations are potentially reversible which makes them attractive and promising targets for therapeutic intervention.

In recent study [1], we utilized methylated DNA immunoprecipitation (MeDIP) and expression microarray to screen the metastasis-associated genes in two syngeneic primary human osteosarcoma cell lines with disparate metastatic potentials. We identified Iroquois homeobox 1 (IRX1) as a candidate pro-metastatic gene that may be activated by DNA methylation. IRX1 is a member of the Iroquois homeobox family of transcription factors which play a crucial role in embryonic development. IRX1 has been previously shown to function as a potential tumor suppressor in gastric cancer [2]; however, its function in cancer development remains largely unknown. Here, we found that IRX1 was upregulated in highly metastatic osteosarcoma cell lines and osteosarcoma tissues from lung metastasis. Gain and loss-of-function assays indicated that IRX1 could enhance the metastatic activity of osteosarcoma cells both in vitro (migration, invasion and resistance to anoikis) and in vivo (murine models). Further study demonstrated that pro-metastatic effects of IRX1 were mediated by upregulation of CXCL14/NF-κB signaling.

On the other hand, IRX1 overexpression in osteosarcoma was strongly associated with hypomethylation of its promoter in both cell lines and clinical tissues. Treatment of ZOS and MNNG/HOS osteosarcoma cells with 5-aza-2′-deoxycytidine (DNA demethylating drug) remarkably decreased the methylation level of the IRX1 promoter and reactivated the IRX1 gene expression. Conversely, treatment of ZOSM and 143B osteosarcoma cells with the methyl donor S-adenosyl-L-methionine (AdoMet, a DNA methylating drug) induced IRX1 promoter methylation and silenced IRX1 expression. Moreover, methylation luciferase assay showed that methylation repressed IRX1 promoter activity in a methylation dose-dependent manner. These data indicated that elevated IRX1 expression in osteosarcoma is associated with hypomethylation of its promoter.

While aberrant hypermethylation commonly happened in the early stage of tumor formation, hypomethylation may occur during the tumor progression. Currently, the majority of studies have focused on promoter hypermethylation of tumor-suppressor genes. The influence of hypomethylation, especially the loss of promoter methylation, has been underestimated. Some prometastatic genes such as S100A4 and Urokinase (uPA) have been found to be activated by promoter hypomethylation in pancreas and breast cancer [3, 4]. Reversal of hypomethylation of these genes may be helpful for anti-metastasis therapy. Indeed, in our study, treatment of 143B cells with AdoMet inhibited the migratory and invasive abilities in vitro and their metastatic potential in vivo. However, we have to keep in mind that DNA methylating drug used in current studies is broadly acting; it may potentially induce hypermethylation of some tumor suppressor genes. Therefore, it is important to develop a more potent and specific methylating drug in the future.

Since aberrant gene methylation is one of the earliest molecular alterations that occur during cancer progression [5], detection of IRX1 hypomethylation could be a promising strategy for the early detection of osteosarcoma metastasis. However, tumor specimens are sometimes difficult to obtain or are unavailable. Circulating tumor DNA fragments (ctDNA), which were released by tumor cells into the blood, contain identical genetic alterations to those in the tumors themselves. Cancer-related molecular changes, such as somatic point mutations, gene copy number variations and loss of heterozygosity, have been detected in the ctDNA. This offers us an opportunity to determine the IRX1 promoter methylation status from the circulation which is more easily available and less invasive. In this study, ctDNA was obtained from the serum of osteosarcoma patients. Using highly sensitive methylation-specific PCR (MSP), we found that the IRX1 promoter was hypomethylated in 46.3% (31 of 67) of serum DNA samples and that patients with IRX1 promoter hypomethylation have a higher risk of developing lung metastasis, suggesting that detection of methylation changes of metastatic genes in the patients' serum DNA could be a potential method to monitor cancer metastasis.

In summary, our work revealed that IRX1 hypomethylation was a predicted marker for osteosarcoma metastasis, and reversion of hypomethylation-activated IRX1 might be beneficial for anti-metastasis therapy.
